# Biorthogonal wavelets and tight framelets from smoothed pseudo splines

**DOI:** 10.1186/s13660-017-1439-3

**Published:** 2017-07-14

**Authors:** Jie Zhou, Hongchan Zheng

**Affiliations:** Department of Applied Mathematics, Northwestern Polytechnical University, Xi’an, Shaanxi 710072 P.R. China

**Keywords:** smoothed pseudo spline, linear independence, biorthogonal wavelet, tight wavelet frame

## Abstract

In order to get divergence free and curl free wavelets, one introduced the smoothed pseudo spline by using the convolution method. The smoothed pseudo splines can be considered as an extension of pseudo splines. In this paper, we first show that the shifts of a smoothed pseudo spline are linearly independent. The linear independence of the shifts of a pseudo spline is a necessary and sufficient condition for the construction of the biorthogonal wavelet system. Based on this result, we generalize the results of Riesz wavelets and derive biorthogonal wavelets from smoothed pseudo splines. Furthermore, by applying the unitary extension principle, we construct tight frame systems associated with smoothed pseudo splines with desired approximation order.

## Introduction

In order to construct tight framelets with desired approximation orders, the first type of pseudo splines was first introduced in [1] and [2]. These pseudo splines are refinable and compactly supported. In general, these pseudo splines are neither symmetric nor antisymmetric. In order to construct symmetric or antisymmetric tight framelets with required approximation orders, Dong introduced the second type of pseudo splines in [3]. The pseudo splines were shown to be an important family of refinable functions. They can provide a wide variety of choices of refinable functions; B-splines, the orthogonal refinable functions and the interpolatory refinable functions are special case of them. Hence, they have large flexibilities in wavelets and framelets construction.

Regarding the pseudo splines, there have been many developments in the theory and applications over the past ten years. Their applications in image denoising and image in-painting are very extensive. The pseudo splines consist of a rich family of compactly supported refinable functions. Together with the unitary extension principle of [4], one gave a range of choices of wavelet systems to meet various demands of applications. Depending on the choice of the parameters, pseudo splines with various orders fill in the gaps between the B-splines and orthogonal refinable functions for the first type and between B-splines and interpolatory refinable functions for the second type. For the subdivision schemes, pseudo splines provide various choices that meet different demands for balancing the approximation power, the length of the support, and the regularity of the limit functions. For the box splines, Li extended the results of [1] and [2], investigated pseudo box splines, and analyzed their properties, including stability and regularity in [5]. Dong showed that the shifts of arbitrarily given pseudo splines are linearly independent in [6]. Bin and Shen in [7] further studied the construction of biorthogonal wavelets from the pseudo splines and derived a dual refinable function with prescribed regularity. The dual pseudo splines are a new family of refinable functions, they were introduced by Dyn and Hormann in [8] as limits of subdivision schemes. The important properties of a dual pseudo spline, such as regularity, stability, and linear independence, were derived in [9]. Yi and Song constructed Riesa wavelets and tight framelets from dual pseudo splines in [10]. Shen and Li introduced complex pseudo splines that were derived from the first type of the pseudo splines in [11], and they analyzed that shifts of every complex pseudo splines are linearly independent. Moreover, they constructed complex Riesz wavelets and complex tight framelets. In order to get divergence free and curl free wavelets, Zhuang and Yang presented smoothed pseudo splines in [12] and discussed the regularity and stability of smoothed pseudo splines.

In this paper, firstly, we show that the shifts of a smoothed pseudo spline are linearly independent. Base on this result, we construct a biorthogonal wavelet system from smoothed pseudo splines and generalize the results of Riesz wavelets in [12]. Moreover, by using the unitary extension principle, we get the construction of tight framelets with desired approximation order based on smoothed pseudo splines.

## Preliminaries

A function $\phi\in L_{2}(\mathbb{R})$ is refinable if it satisfies the following refinement equation:

2.1 $$ \phi=2\sum_{k\in\mathbb{Z}}a(k)\phi(2\cdot-k), $$

where *a* is a finitely supported sequence on $\mathbb{Z}$, called the refinement mask of the refinement function *ϕ*. The Fourier transform of a function $f\in L_{1}(\mathbb{R})$ is defined by

$$ \hat{f}(\xi)= \int_{\mathbb{R}}f(x)e^{-ix \xi}\,dx, \quad\xi\in \mathbb{R}, $$

and it can be naturally extended to $L_{2}(\mathbb{R})$ functions. The Fourier series of a sequence *a* on $\mathbb{Z}$ is defined by

$$ \hat{a}(\xi)=\sum_{k\in\mathbb{Z}}a(k)e^{-i\xi k}, \quad \xi\in \mathbb{R}. $$

In terms of the Fourier transform, the refinement equation in (2.1) can be rewritten as

$$ \hat{\phi}(\xi)=\hat{a} \biggl(\frac{\xi}{2} \biggr)\hat{\phi } \biggl( \frac{\xi}{2} \biggr), \quad\xi\in\mathbb{R}. $$

We call *â* a refinement mask for convenience. It may also be given in terms of its symbol $\tilde{a}(z)=\sum_{k\in\mathbb{Z}}a(k)z^{k}$, $z\in\mathbb{C}\setminus\{0\}$, which relates to the corresponding Fourier series by $\hat{a}(\xi)=\tilde{a}(e^{-i\xi})$, $\xi\in\mathbb{R}$. Pseudo splines are defined in terms of their refinement masks. The refinement mask of the first type of pseudo splines with order $(m,l)$ is given by

2.2 $$ \big|{}_{1}\hat{a}(\xi)\big|^{2}=\big|{}_{1} \hat{a}_{(m,l)}(\xi)\big|^{2}=\cos^{2m} \biggl( \frac{\xi}{2} \biggr)\sum_{j=0}^{l} {m+l\choose j}\sin^{2j} \biggl(\frac{\xi}{2} \biggr)\cos ^{2(l-j)} \biggl(\frac{\xi}{2} \biggr), $$

and the refinement mask of the second type of pseudo splines with order $(m,l)$ is given by

2.3 $$ _{2}\hat{a}(\xi)= {_{2}\hat{a}_{(m,l)}}( \xi)=\cos^{2m} \biggl(\frac {\xi}{2} \biggr)\sum _{j=0}^{l} {m+l\choose j}\sin^{2j} \biggl(\frac{\xi}{2} \biggr)\cos ^{2(l-j)} \biggl(\frac{\xi}{2} \biggr), $$

where $0\leq l\leq{m-1}$.

According to the Fejér-Riesz lemma (see, e.g., [13] and [14]), we have that the mask of the first type of pseudo splines is obtained by taking the square root of the mask of the second type of pseudo splines, i.e., $_{2}\hat{a}(\xi )=|{}_{1}\hat{a}(\xi)|^{2}$.

The corresponding pseudo splines can be defined in terms of their Fourier transforms as

$$ _{k}\hat{\phi}_{(m,l)}(\xi)= \prod _{j=1}^{\infty} {_{k}\hat {a}_{(m,l)} \bigl(2^{-j}\xi\bigr)}, \quad k=1,2, $$

with $_{k}\hat{\phi}_{(m,l)}(0)=1$.

The smoothed pseudo splines were introduced in [12] in order to smoothen the pseudo splines by using the convolution method. The smoothed pseudo splines are defined in terms of their refinement masks. One can take the smoothed pseudo spline

$$ \phi_{n,m,l}=\phi_{m,l}\ast\underbrace{ \chi_{[-\frac{1}{2},\frac{1}{2}]}\ast \cdots\ast\chi_{[-\frac {1}{2},\frac{1}{2}]}}_{n-m}, $$

where $\chi_{[-\frac{1}{2},\frac{1}{2}]}$ denotes the characteristic function of interval $[-\frac{1}{2},\frac{1}{2}]$, and $n\geq m$. The refinement mask of a smoothed pseudo spline of type I with order $(n,m,l)$ is given by

2.4 $$ \big|{}_{1}\hat{a}_{n,m,l}(\xi)\big|^{2}= \cos^{2n} \biggl(\frac{\xi}{2} \biggr)\sum _{j=0}^{l} {m+l\choose j}\sin^{2j} \biggl(\frac{\xi}{2} \biggr)\cos ^{2(l-j)} \biggl(\frac{\xi}{2} \biggr). $$

The refinement mask of a smoothed pseudo spline of type II with order $(r,m,l)$ is given by

2.5 $$ _{2}\hat{a}_{r,m,l}(\xi)=\cos^{r} \biggl(\frac{\xi}{2} \biggr)\sum_{j=0}^{l} {m+l\choose j}\sin^{2j} \biggl(\frac{\xi}{2} \biggr)\cos ^{2(l-j)} \biggl(\frac{\xi}{2} \biggr), $$

where $r\geq2m$. The smoothed pseudo splines can be considered as an extension of the pseudo splines. When $r=2m$, they are the pseudo splines.

The smoothed dual pseudo splines are also an extension of the dual pseudo splines. Similar to the definition of (2.4), we can get the smoothed dual pseudo splines. Their refinement mask is given by

$$ \hat{b}_{n,m,l}(\xi)=e^{i \frac{\xi}{2}}\cos^{2n+1} \biggl(\frac {\xi}{2} \biggr)\sum_{j=0}^{l} {m+l+1/2\choose j}\sin^{2j} \biggl(\frac{\xi}{2} \biggr)\cos ^{2(l-j)} \biggl(\frac{\xi}{2} \biggr). $$

Furthermore, we assume $n\in\mathbb{R}$ in (2.4), then the smoothed dual pseudo splines are generalized to the fractional splines in [15]. If we define the translated form of the type II by $_{T}\hat{\phi}_{r,m,l}(\xi)=e^{-i r \frac{\xi}{2}}{_{2}\hat{\phi }_{r,m,l}}(\xi)$, we can get the differential relation $_{T}\phi'_{{r+1},m,l}(x)={_{T}\phi _{r,m,l}}(x)-{_{T}\phi_{r,m,l}(x-1)}$, which plays an important role in the construction of divergence free wavelets and curl free wavelets in the analysis of incompressible turbulent flows. For more details on the construction of divergence free and curl free wavelets, see [16] and [17].

We now define the following functions:

$$ \begin{gathered} P_{m,l}(y)=\sum_{j=0}^{l} {m+l\choose j}y^{j}(1-y)^{l-j},\\ R_{m,l}(y)=(1-y)^{m} P_{m,l}(y),\\ R_{r,m,l}(y)=(1-y)^{\frac{r}{2}} P_{m,l}(y), \end{gathered} $$

denoting $y=\sin^{2} (\frac{\xi}{2} )$, *r*, *m*, *l* are nonnegative integers and $r\geq2m$. Then we can find that

$$ R_{m,l} \biggl(\sin^{2} \biggl(\frac{\xi}{2} \biggr) \biggr)={_{2}\hat {a}_{m,l}} \biggl(\frac{\xi}{2} \biggr), \qquad R_{r,m,l} \biggl(\sin^{2} \biggl(\frac{\xi}{2} \biggr) \biggr)={_{2}\hat {a}_{r,m,l}}(\xi). $$

We now give the following three lemmas to prove the key results of this paper.

### Lemma 2.1

[3]

*For nonnegative integers* *m* *and* *l* *with* $l\leq m-1$, *let* $R_{m,l}$ *and* $P_{m,l}$ *be the polynomials defined above*. *Then*

1. $P_{m,l}(y)=\sum_{j=0}^{l}{m-1+j\choose j}y^{j}$;
2. $R'_{m,l}(y)=-(m+l){m+l-1\choose l}y^{l}(1-y)^{m-1}$;
3. $R'_{r,m,l}(y)=-(\frac{r}{2}-m)(1-y)^{\frac {r}{2}-m-1}R_{m,l}(y)+(1-y)^{\frac{r}{2}-m}R'_{m,l}(y)$.

### Lemma 2.2

[12]

*For nonnegative integers* *m*, *r* *and* *l*.

1. *Define* $Q(y)=R_{r,m,l}(y)+R_{r,m,l}(1-y)$, *then*
  $$ \min _{y\in[0,1]}Q(y)=Q \biggl(\frac{1}{2} \biggr)=2^{1-\frac {r}{2}-l} \sum_{j=0}^{l}{m+l\choose j}. $$
2. *Define* $S(y)=R^{2}_{r,m,l}(y)+R^{2}_{r,m,l}(1-y)$, *then*
  $$ \min _{y\in[0,1]}S(y)=S \biggl(\frac{1}{2} \biggr)=2^{1-r-2l} \Biggl(\sum_{j=0}^{l}{m+l\choose j} \Biggr)^{2}. $$

### Lemma 2.3

[3]

*For given nonnegative integers* *m*, *r* *and* *l* *with* $m\geq1$, $0 \leq l \leq{m-1}$, *we have*

$$ 2^{l} {m+l\choose l}^{\frac{1}{2}}\leq\sum _{j=0}^{l}{m+l\choose j}. $$

## Linear independence of smoothed pseudo splines

The section is devoted to analyzing linear independence of the shifts of smoothed pseudo splines, which is a necessary and sufficient condition for the existence of the biorthogonal wavelets. It ensures the existence of the biorthogonal dual refinable function with arbitrarily prescribed regularity. This is stronger than the statement that the shifts of a smoothed pseudo spline form a Riesz system, see [18].

We said that the shifts of a compactly supported function are linearly independent if and only if

$$ \sum_{j\in\mathbb{Z}}a(j)\phi(\cdot-j)=0\quad\Longrightarrow \quad a=0, $$

for all sequences $a\in l_{2}\{\mathbb{Z}\}$, $\phi\in L_{2}(\mathbb{R})$.

We get the following lemma about the linear independence of the shifts of a compactly supported function.

### Lemma 3.1

[6] *Let* $\phi\in L_{2}(\mathbb{R})$ *be a compactly supported refinable function with finitely supported refinement mask* *a*. *The shifts of* *ϕ* *are linearly independent if and only if*:

1. *ϕ* *is stable*;
2. *the symbol* *ã* *does not have any symmetric zeros on* $\mathbb{C}\setminus\{0\}$.

We are now ready to discuss the linear independence of the shifts of the smoothed pseudo splines. According to Lemma 3.1, in order to show the linear independence of the shifts of the smoothed pseudo splines, we need to verify that (i) the smoothed pseudo splines are stable, (ii) the symbol of an arbitrary smoothed pseudo spline does not have any symmetric zeros on $\mathbb{C}\setminus\{0\}$. The stability of the smoothed pseudo splines was shown in [12], we only need to verify condition (ii). We have the following theorem.

### Theorem 3.1

*The shifts of any smoothed pseudo splines of type II are linearly independent*.

### Proof

The refinement mask of a smoothed pseudo spline of type II with order $(r,m,l)$ is given by (2.5), i.e.,

$$ _{2}\hat{a}_{r,m,l}(\xi)=\cos^{r} \biggl(\frac{\xi}{2} \biggr)\sum_{j=0}^{l} {m+l\choose j}\sin^{2j} \biggl(\frac{\xi}{2} \biggr)\cos ^{2(l-j)} \biggl(\frac{\xi}{2} \biggr). $$

Using $\cos^{2} (\frac{\xi}{2} )=\frac{(1+e^{-i\xi })^{2}}{4e^{-i\xi}}$, $\sin^{2} (\frac{\xi}{2} )=\frac {-(1-e^{-i\xi})^{2}}{4e^{-i\xi}}$ in Eq. (2.5), we can get

$$ _{2}\hat{a}_{(r,m,l)}(\xi)=\frac{(1+e^{-i\xi})^{r}}{(4e^{-i\xi })^{\frac{r}{2}}}\sum _{j=0}^{l}{m+l\choose j} \biggl( \frac{-(1-e^{-i\xi})^{2}}{4e^{-i\xi}} \biggr)^{j} \biggl(\frac {(1+e^{-i\xi})^{2}}{4e^{-i\xi}} \biggr)^{l-j}. $$

Substituting $z=e^{-i\xi}$, the symbol of the smoothed pseudo splines of type II can be written as

$$\begin{aligned} _{2}\tilde{a}(z) &= \frac{(1+z)^{r}}{(4z)^{\frac{r}{2}}}\sum _{j=0}^{l}{m+l\choose j} \biggl( \frac{-(1-z)^{2}}{4z} \biggr)^{j} \biggl(\frac{(1+z)^{2}}{4z} \biggr)^{l-j} \\ &=\frac{(1+z)^{r}}{(4z)^{\frac{r}{2}+l}}\sum_{j=0}^{l} {m+l\choose j}\bigl(-(1-z)^{2}\bigr)^{j}(1+z)^{2(l-j)} \\ &=\frac{(1+z)^{r+2l}}{(4z)^{\frac{r}{2}+l}}\sum_{j=0}^{l} {m+l\choose j} \biggl(\frac{-(1-z)^{2}}{(1+z)^{2}} \biggr)^{j}. \end{aligned}$$

Obviously, $z=-1$ is a zero of ${_{2}\tilde{a}}(z)$, when $z=1$, ${_{2}\tilde{a}}(1)=1$. Hence, $_{2}\tilde{a}(z)$ having no symmetric zeros on $\mathbb{C}\setminus\{ 0\}$ is equivalent to the polynomial

$$ p(z)=\sum_{j=0}^{l}{m+l\choose j} \biggl(\frac{-(1-z)^{2}}{(1+z)^{2}} \biggr)^{j} $$

having no symmetric zeros on $\mathbb{C} \setminus\{0,1,-1\}$.

Consider

$$ h(x)=\sum_{j=0}^{l} h_{j} x^{j}, $$

with $h_{j}={m+l\choose j}$, $x\in\mathbb{C}$. Since coefficients $h_{j}$ of the polynomial $p(z)$ form a strictly positive and increasing sequences, using Proposition 2.3 of [6], we can have that all zeros of any polynomial $h(x)=\sum_{j=0}^{l} h_{j}x^{j}$ are contained in the open unit disk $D:=\{x\in\mathbb{C}:|x|<1\}$. Suppose $p(z)$ has symmetric zeros $z_{0}$ and $-z_{0}$. Then they must both be in *D*, therefore,

$$ |x_{1}||x_{0}|< 1. $$

Since

$$ |x_{0}|= \bigg|-\frac{(1-z_{0})^{2}}{(1+z_{0})^{2}} \bigg|=\frac{1}{ |-\frac {(1+z_{0})^{2}}{(1-z_{0})^{2}} |}= \frac{1}{|x_{1}|}, $$

we conclude that $|x_{1}||x_{0}|=1$, which contradicts $|x_{1}||x_{0}|<1$. It follows that $p(z)$ has no symmetric zeros on $\mathbb{C} \setminus\{0,1,-1\}$. Hence, $_{2}\tilde {a}(z)$ has no symmetric zeros on $\mathbb{C} \setminus\{0\}$. Together with the stability of smoothed pseudo splines, we conclude the proof. □

## The construction of biorthogonal wavelet

In this section, based on the results of Section 3, we focus on the construction of biorthogonal wavelets from the smoothed pseudo splines. The biorthogonal wavelets are a general form of Riesz wavelets. Firstly, we begin with some basics. For a given $\psi\in L_{2}(\mathbb{R})$, define the wavelet system

$$ X(\psi):=\bigl\{ \psi_{n,k}=2^{n/2}\psi\bigl(2^{n} \cdot-k\bigr):n,k\in\mathbb{Z}\bigr\} . $$

If, for some $C_{1}>0$ and for every $f\in L_{2}(\mathbb{R})$, we have

$$ \sum_{g\in X(\psi)} \big|\langle f,g\rangle\big|^{2} \leq C_{1} \| f \|^{2}_{L_{2}(\mathbb{R})}, $$

then we call system $X(\psi)$ a Bessel system. A Bessel system $X(\psi)$ is a Riesz basis for $L_{2}(\mathbb{R})$ if there exists $C_{2}>0 $ such that

$$ C_{2}\big\| \{c_{n,k}\}\big\| _{l_{2}(\mathbb{Z}^{2})}\leq \bigg\| \sum_{(n,k)\in\mathbb{Z}^{2}} c_{n,k}\psi_{n,k} \bigg\| _{L_{2}(\mathbb{R})} $$

for all $\{c_{n,k}\}\in l_{2}{(\mathbb{Z}^{2})}$, and the span of $\{\psi _{n,k}\in\mathbf{}\mathbb{Z}\}$ is dense in $L_{2}(\mathbb{R})$. We call the function *ψ* a Riesz wavelet. If $X(\psi)$ forms a Riesz basis for $L_{2}(\mathbb{R})$, $X(\psi)$ is also called the Riesz wavelet system.

Let a refinable function $\phi\in L_{2}(\mathbb{R}) $ be a compactly supported orthonormal refinable function satisfying the following refinement equation:

$$ \phi=2\sum_{k\in\mathbb{Z}}a_{k}\phi(2 \cdot-k), $$

one can easily obtain a wavelet function *ψ* by

4.1 $$ \psi=2\sum_{k\in\mathbb{Z}}(-1)^{k-1} \overline{a(1-k)}\phi (2\cdot-k). $$

It is well known that *ψ* generates an orthonormal wavelet basis for $L_{2}(\mathbb{R})$. We are interested in knowing whether the function *ψ* defined in (4.1) is a Riesz wavelet when the refinable function *ϕ* is chosen to be different refinable functions. When the refinable function *ϕ* is a B-spline, Han showed in [19] that the wavelet defined in (4.1) is a Riesz wavelet. When the refinable function *ϕ* is chosen to be a pseudo spline, Dong in [3] gave the same results. If the refinable function *ϕ* is a smoothed pseudo spline, Zhuang obtained the following results.

### Theorem 4.1

[12]

*Let*_*k*_ *ϕ*, $k=1,2$, *be the smoothed pseudo splines of types I and types II with order* $(r,n,m,l)$. *The refinement masks*_*k*_ *a* *are given in* (2.4) *and* (2.5). *Define*

$$ _{k}\hat{\psi}(2\xi)=e^{-i\xi} \overline{_{k}\hat{a}(\xi+\pi)} _{k}\hat{\phi}(\xi), \quad k=1,2. $$

*Then* $X(\psi)$ *is a Riesz basis for* $L_{2}(\mathbb{R})$.

Now, we give the main results in this section. We start with a lemma and a corollary.

### Lemma 4.1

[19]

*Let* *a* *and* *b* *be two sequences on* $\mathbb{Z}$ *satisfying the following two conditions*:

(i) $$ \big|\hat{a}(\xi) \big|= \biggl(\frac{1+e^{-i\xi}}{2} \biggr)^{m} \hat {A}(\xi); \qquad \hat{b}(\xi)= \biggl(\frac{1-e^{i\xi}}{2} \biggr)^{\tilde{m}} \hat {B}( \xi), $$
  *where* *m* *and* *m̃* *are positive integers*, *A* *and* *B* *are sequences on* $\mathbb{Z}$ *with polynomial decay satisfying* $\hat{A}(0)=1$ *and* $\hat{B}(\pi )\neq0$.
(ii) *Let*
  $$ \hat{\tilde{a}}(\xi)= \biggl(\frac{1+e^{-i\xi}}{2} \biggr)^{\tilde {m}} \hat{ \tilde{A}}(\xi), \qquad \hat{\tilde{b}}(\xi)=\frac{\overline{\hat{a}(\xi+\pi )}}{\overline{\hat{d}(\xi)}}, $$
  *where* $\hat{\tilde{A}}(\xi)=\frac{\overline{\hat{B}(\xi+\pi )}}{\overline{\hat{d}(\xi)}}$, $\hat{d}(\xi)=\hat{a}(\xi)\hat{b}(\xi+\pi)-\hat{a}(\xi +\pi)\hat{b}(\xi)\neq0$, *for all* $\xi\in\mathbb{R}$.

*Define*

$$\begin{gathered} \hat{\phi}(\xi)=\sum_{j=1}^{\infty}\hat{a} \bigl(2^{-j}\xi\bigr),\qquad\hat {\tilde{\phi}}(\xi)=\sum _{j=1}^{\infty}\hat{\tilde{a}}\bigl(2^{-j}\xi \bigr), \\ \hat{\psi}(\xi)= \hat{b} \biggl(\frac{\xi}{2} \biggr)\hat{\phi } \biggl( \frac{\xi}{2} \biggr),\qquad\hat{\tilde{\psi}}(\xi)=\hat {\tilde{b}} \biggl( \frac{\xi}{2} \biggr)\hat{\tilde{\phi}} \biggl(\frac{\xi}{2} \biggr). \end{gathered}$$

*Assume that*

$$ \limsup _{n\rightarrow\infty}{\| A_{n}\| }^{1/n}_{l_{2}(\mathbb{Z})}< 2^{m-\frac{1}{2}}, \qquad \limsup _{n\rightarrow\infty}{\|\tilde{A}_{n}\| }^{1/n}_{l_{2}(\mathbb{Z})}< 2^{\tilde{m}-\frac{1}{2}}, $$

*where*

4.2 $$ \hat{A}_{n} {(\xi)}=\hat{A}\bigl(2^{n-1}\xi \bigr)\cdots\hat{A}(2\xi)\hat {A}(\xi),\qquad \hat{\tilde{A}}_{n} {( \xi)}=\hat{\tilde{A}}\bigl(2^{n-1}\xi\bigr)\cdots\hat {\tilde{A}}(2\xi) \hat{\tilde{A}}(\xi). $$

*Then all the functions* *ϕ*, *ϕ̃*, *ψ*, *ψ̃* *belonging to* $L_{2}(\mathbb{R})$ *satisfy*

$$\begin{gathered} \bigl\langle \phi,\tilde{\phi}(\cdot-k)\bigr\rangle =\bigl\langle \psi,{\tilde { \psi}(\cdot-k)} \bigr\rangle =\delta(k), \\ \bigl\langle \phi,\tilde{\psi}(\cdot-k)\bigr\rangle =\bigl\langle \psi,{\tilde { \phi}(\cdot-k)} \bigr\rangle =0, \quad k \in\mathbb{Z}. \end{gathered}$$

With this, Han gave the following corollary as a direct result of Lemma 4.1.

### Corollary 4.1

[19]

*Let the sequences* *a* *and* *b* *be given in Lemma* 4.1, *and the sequences* *ã*, *b̃*, *A*, *Ã* *and the functions* *ϕ*, *ϕ̃*, *ψ*, *ψ̃* *belonging to* $L_{2}(\mathbb{R})$ *be defined as in Lemma* 4.1. *Define*

$$ \rho_{A}=\inf _{n\in\mathbb{N}} \|\hat{A}_{n} \| ^{1/n}_{L_{\infty}(\mathbb{R})} , \qquad \rho_{\tilde{A}}=\inf _{n\in\mathbb{N}} \| \hat {\tilde{A}}_{n} \| ^{1/n}_{L_{\infty}(\mathbb{R})}, $$

*where* $A_{n}$ *and* $\tilde{A}_{n}$ *are defined in* (4.2). *Then*, *for any* $\varepsilon>0$, *there exists a positive constant* *M* *such that*

$$\begin{gathered} \max \bigl( \big|{\hat{\phi}(\xi)} \big|, \big|{\hat{\psi}(\xi )} \big| \bigr)\leq M(1+| \xi|)^{-m+\varepsilon+\log_{2}{\rho_{A}}}, \quad\forall \xi\in\mathbb{R}, \\ \max \bigl( \big|\hat{\tilde{\phi}}(\xi) \big|, \big|\hat{\tilde {\psi}}(\xi) \big| \bigr)\leq M(1+|\xi|)^{{-\tilde{m}}+\varepsilon +\log_{2}{\rho_{\tilde{A}}}}, \quad\forall \xi \in\mathbb{R}. \end{gathered}$$

*Consequently*, *if* $\rho_{A}<2^{m-{\frac{1}{2}}}$ *and* $\rho_{\tilde {A}}<2^{\tilde{m}-{\frac{1}{2}}}$, *then* $(X(\psi),X(\tilde{\psi}))$ *forms a pair of biorthogonal wavelet bases in* $L_{2}(\mathbb{R})$. *In particular*, $X(\psi)$ *is a Riesz basis of* $L_{2}(\mathbb{R})$.

With Lemma 4.1 and Corollary 4.1, we prove the following result on the biorthogonal wavelets infinite masks from smoothed pseudo splines.

### Theorem 4.2

*Let*_*k*_ *ϕ*, $k=1,2$, *be the smoothed pseudo splines of types I and types II with order* $(r,n,m,l)$, *the refinement masks*_*k*_ *â* *are given in* (2.4) *and* (2.5). *Let* *b* *be a sequence on* $\mathbb{Z}$, *if* *â*, *b̂* *can be factorized into the form*

$$ \big|\hat{a}(\xi) \big|= \biggl(\frac{1+e^{-i\xi}}{2} \biggr)^{m} \hat {A}(\xi); \qquad \hat{b}(\xi)= \biggl(\frac{1-e^{i\xi}}{2} \biggr)^{\tilde{m}} \hat {B}( \xi). $$

*Assume*

$$ \hat{\tilde{a}}(\xi)= \biggl(\frac{1+e^{-i\xi}}{2} \biggr)^{\tilde {m}} \hat{ \tilde{A}}(\xi), \qquad \hat{\tilde{b}}(\xi)=\frac{\overline{\hat{a}(\xi+\pi )}}{\overline{\hat{d}(\xi)}}, $$

*where* $\hat{\tilde{A}}(\xi)=\frac{\overline{\hat{B}(\xi+\pi )}}{\overline{\hat{d}(\xi)}}$, $\hat{d}(\xi)=\hat{a}(\xi)\hat{b}(\xi+\pi)-\hat{a}(\xi +\pi)\hat{b}(\xi)$.

*Define*

$$\begin{gathered} \hat{\phi}(\xi)=\sum_{j=1}^{\infty}\hat{a} \bigl(2^{-j}\xi\bigr),\qquad\hat {\tilde{\phi}}(\xi)=\sum _{j=1}^{\infty}\hat{\tilde{a}}\bigl(2^{-j}\xi \bigr), \\ \hat{\psi}(\xi)=\hat{b} \biggl(\frac{\xi}{2} \biggr)\hat{\phi } \biggl( \frac{\xi}{2} \biggr),\qquad\hat{\tilde{\psi}}(\xi)=\hat {\tilde{b}} \biggl( \frac{\xi}{2} \biggr)\hat{\tilde{\phi}} \biggl(\frac{\xi}{2} \biggr). \end{gathered}$$

*If we have*

$$ \limsup _{n\rightarrow\infty}{\| A_{n}\| }^{1/n}_{l_{2}(\mathbb{Z})}< 2^{m-\frac{1}{2}}, \qquad \limsup _{n\rightarrow\infty}{\|\tilde{A}_{n}\| }^{1/n}_{l_{2}(\mathbb{Z})}< 2^{\tilde{m}-\frac{1}{2}}, $$

*where*

$$ \hat{A}_{n} {(\xi)}=\hat{A}\bigl(2^{n-1}\xi \bigr)\cdots\hat{A}(2\xi)\hat {A}(\xi),\qquad \hat{\tilde{A}}_{n} {( \xi)}=\hat{\tilde{A}}\bigl(2^{n-1}\xi\bigr)\cdots\hat {\tilde{A}}(2\xi) \hat{\tilde{A}}(\xi), $$

*then*

$$\begin{gathered} \bigl\langle \phi,\tilde{\phi}(\cdot-k)\bigr\rangle =\bigl\langle \psi,{\tilde { \psi}(\cdot-k)} \bigr\rangle =\delta(k), \\ \bigl\langle \phi,\tilde{\psi}(\cdot-k)\bigr\rangle =\bigl\langle \psi,{\tilde { \phi}(\cdot-k)} \bigr\rangle =0, \quad k \in\mathbb{Z}. \end{gathered}$$

### Proof

We first note that

$$\begin{gathered} \big|{_{1}{\hat{a}_{n,m,l}}(\xi)} \big|^{2}+ \big|{_{1} \hat {a}_{n,m,l}(\xi+\pi)} \big|^{2}= R_{2n,m,l}(y)+R_{2n,m,l}(1-y) \neq0; \\ \big|{_{2}{\hat{a}_{r,m,l}}(\xi)} \big|^{2}+ \big|{_{2} \hat {a}_{r,m,l}(\xi+\pi)} \big|^{2}= R^{2}_{r,m,l}(y)+R^{2}_{r,m,l}(1-y) \neq0, \\\begin{aligned} \hat{d}(\xi)&=\hat{a}(\xi)\hat{b}(\xi+\pi)-\hat{a}(\xi+\pi )\hat{b}(\xi) \\ &= e^{-i(\xi+\pi)} \bigl( \big|\hat{a}(\xi) \big|^{2}+ \big|\hat {a}(\xi+\pi) \big|^{2} \bigr)\neq0, \quad\xi\in[-\pi,\pi]. \end{aligned}\end{gathered}$$

According to Theorem 4.1, when _*k*_ *ϕ*, $k=1,2$, are the smoothed pseudo splines, $_{k}\hat{\psi}(2\xi)=e^{-i\xi}\overline{_{k}\hat{a}(\xi+\pi)} _{k}\hat{\phi}(\xi)$ is a Riesz wavelet, $X(\psi)$ is a Riesz basis for $L_{2}(\mathbb{R})$. We have

$$\begin{gathered} \big|{_{1}{\hat{a}_{n,m,l}}(\xi)}\big|^{2}={_{2}{{ \hat {a}_{2n,m,l}}}}(\xi)= \biggl(\frac{1+e^{-i\xi}}{2} \biggr)^{2n} \hat {A}(\xi), \\ \hat{b}(\xi)=e^{-i\xi}\overline{_{k}\hat{a}(\xi+ \pi)}=e^{-i\xi } \biggl(\frac{1+e^{i\xi}}{2} \biggr)^{2n} \overline{ \hat{A}(\xi+\pi)}. \end{gathered}$$

So,

$$ \hat{B}(\xi)=e^{-i\xi}\overline{\hat{A}(\xi+\pi)}, $$

where $\hat{A}(\xi)=\sum_{j=0}^{l} {m+l\choose j}\sin^{2j}(\frac{\xi}{2})\cos^{2(l-j)}(\frac{\xi}{2})$. Next, we check whether $\rho_{A}<2^{m-{\frac{1}{2}}}$ and ${\rho_{\tilde{A}}<2^{\tilde {m}-{\frac{1}{2}}}}$ hold. By the proof of Theorem 4.1, we get

$$ \rho_{A}=\inf \|\hat{A}_{n} \| ^{1/n}_{L_{\infty }(\mathbb{R})} \leq \big\| {\hat{A}_{n}(\xi)} \big\| _{L_{\infty}(\mathbb {R})}\leq2^{n-{\frac{1}{2}}}. $$

Denote $y=\sin^{2}(\frac{\xi}{2})$, we have

$$ _{1}\hat{\tilde{A}}(\xi)=\frac{\overline{\hat{B}(\xi+\pi )}}{\overline{\hat{d}(\xi)}}= \frac{_{1}\hat{A}(\xi)}{R_{2n,m,l}(y)+R_{2n,m,l}(1-y)}. $$

So

$$\begin{aligned} \rho_{{}_{1}\tilde{A}}&= \inf \| {_{1}\hat{\tilde{A}}_{n}} \| ^{1/n}_{L_{\infty}(\mathbb{R})}\leq \sup _{y \in[0,1]} \bigg|\frac{_{1}\hat{A}(\xi )}{R_{2n,m,l}(y)+R_{2n,m,l}{(1-y)}} \bigg| \\ &= \sup _{y \in[0,1]} \bigg|\frac {(P_{m,l}(y))^{1/2}}{R_{2n,m,l}(y)+R_{2n,m,l}{(1-y)}} \bigg| \\ &\leq\frac{{m+l\choose l}^{1/2}}{\min _{y \in [0,1]}(R_{2n,m,l}(y)+R_{2n,m,l}{(1-y)})} \\ &= \frac{2^{n+l-1}{m+l\choose l}^{1/2}}{\sum_{j=0}^{l}{m+l\choose j}}. \end{aligned}$$

Applying Lemma 2.3, we have $\rho_{{}_{1}\tilde{A}}=\inf \|{}_{1}{\hat{\tilde{A}}_{n}} \| ^{1/n}_{L_{\infty}(\mathbb{R})}\leq2^{n-\frac{1}{2}}$. Similarly, we also obtain $\rho_{{}_{2}\tilde{A}}=\inf \|{}_{2}{\hat{\tilde{A}}_{r}} \|^{1/r}_{L_{\infty}(\mathbb{R})}\leq2^{r-\frac{1}{2}}$. This completes the proof. □

## The construction of tight framelets

In this section, we give a construction of tight framelets and discuss the approximation order of tight framelets from smoothed pseudo splines by using the unitary extension principle of [4]. The construction here is based on the unitary extension principle, we make use of the pseudo splines of type II to obtain a tight frame. Before proceeding further, let us recall some basic definitions.

A system $X(\Psi)$ with $\Psi=\{\psi^{1},\ldots, \psi^{L}\}$ is a frame for $L_{2}(\mathbb{R})$ if there exist positive constants $C_{1}$ and $C_{2}$ such that

$$ C_{1} \| f\|^{2}\leq\sum _{l=1}^{L}\sum_{n\in\mathbb {Z}}\sum _{k\in\mathbb{Z}} \big|\bigl\langle f,\psi^{l}_{n,k} \bigr\rangle \big|^{2}\leq C_{2}\| f\|^{2}, \quad\forall f\in L_{2}(\mathbb{R}), $$

where $\langle f,g\rangle=\int_{\mathbb{R}}f(t)\overline{g(t)}\,dt$, $\| f\|^{2}=\langle f,f\rangle$.

Let $\Psi=\{\psi^{1},\ldots, \psi^{L}\}$ and $\tilde{\Psi}=\{\tilde {\psi}^{1},\ldots, \tilde{\psi}^{L}\}$ be two sets of functions in $L_{2}(\mathbb{R})$ if each of $X(\Psi)$ and $X(\tilde{\Psi})$ is a Bessel system in $L_{2}(\mathbb{R})$ satisfying

5.1 $$ \langle f,g\rangle=\sum_{l=1}^{L} \sum_{n\in\mathbb{Z}}\sum_{k\in \mathbb{Z}} \bigl\langle f,\tilde{\psi}^{l}_{n,k}\bigr\rangle \bigl\langle \psi^{l}_{n,k}, g\bigr\rangle ,\quad\forall f,g\in L_{2}(\mathbb{R}). $$

We say that $(X(\Psi),X(\tilde{\Psi}) )$ is a pair of bi-frames $L_{2}(\mathbb{R})$.

If (5.1) holds with $\tilde{\psi}^{l}=\psi^{l}$ for all $l=1,2,\ldots, L$, then $X(\Psi)$ is a tight wavelet frame in $L_{2}(\mathbb{R})$. If $(X(\Psi ),X(\tilde{\Psi}) )$ satisfies $\langle\psi^{l}_{n,k}, \psi^{l'}_{n',k'}\rangle= \delta(l-l')\delta(n-n')\delta(k-k')$, $n,n',k,k'\in\mathbb{Z}$, for all $l,l'=1,2,\ldots ,L$, then $(X(\Psi),X(\tilde{\Psi}) )$ forms a pair of biorthogonal wavelet bases in $L_{2}(\mathbb{R})$.

For $X(\Psi)$, define the truncated operator as

$$ \mathfrak{H}_{n}: f \rightarrow\sum _{\psi_{i}\in\Psi,k \in \mathbb{Z},j< n} \langle f, \psi_{j,k} \rangle\psi_{j,k}, \quad i=1,2,\ldots, L. $$

We say that the operator $\mathfrak{H}_{n} $ provides approximation order $m_{1}$ if for all *f* in the Sobolev space $W^{m_{1}}_{2}(\mathbb{R})$

$$ \| f-{\mathfrak{H}_{n}}f \|_{2}= O \bigl(2^{-n{m_{1}}}\bigr). $$

Recall that a function *ϕ* satisfies the Strang-Fix (SF) condition of order $m_{0}$ if

$$ \hat{\phi}(0)\neq0, \qquad\hat{\phi}^{(j)}(2\pi k)=0, \quad j=0,1, \ldots,m_{0}-1, k \in\mathbb{Z}\setminus \{0\}. $$

If the refinable function *ϕ* satisfies the Strang-Fix (SF) condition of order $m_{0}$ and the corresponding mask *â* satisfies $1- |\hat{a} |^{2}= O (|\cdot|^{m_{2}} )$ at the origin, then $m_{1}=\min\{m_{0},m_{2}\}$.

Let *â* be the refinement mask of $\phi\in L_{2}(\mathbb{R})$ with $\hat{a}(0)=1$, and the corresponding wavelet masks $\hat{b}_{j}$, $j=1,2,\ldots , L$. If *â*, $\hat{b}_{j}$ are trigonometric polynomials that satisfy

5.2 $$ \big|\hat{a}(\xi) \big|^{2}+\sum_{j=1}^{L} \big|\hat{b}_{j}(\xi) \big|^{2}=1, \qquad\hat{a}(\xi)\overline{ \hat{a}(\xi+\pi)}+\sum_{j=1}^{L}\hat {b}_{j}(\xi)\overline{\hat{b}_{j}(\xi+\pi)}=0, $$

for all $\xi\in[-\pi,\pi]$, and $\Psi=(\psi^{1},\psi^{2},\ldots, \psi^{L})\subset L_{2}(\mathbb{R})$ are given by

$$ \hat{\psi}^{j}(2\xi)=\hat{b}_{j}(\xi)\hat{\phi}(\xi), \quad j=1,2,\ldots,L, $$

then the unitary extension principle asserts that $X(\Psi)$ is a tight frame for $L_{2}(\mathbb{R})$.

### Theorem 5.1

*Let*_2_ *ϕ* *be a smoothed pseudo spline of type II with order* $(r,m,l)$ *and*_2_ *â* *be its refinement mask*. *Suppose*

$$ T:=1- \big|{}_{2}\hat{a}(\xi) \big|^{2}- \big|{}_{2}\hat{a}(\xi+ \pi) \big|^{2}, \qquad\gamma:=\frac{\sqrt{T}}{2}, $$

*where* *γ* *is obtained via the Fejér*-*Riesz lemma*. *Define*

$$ \hat{b}_{1}(\xi)=e^{-i\xi}{}_{2}\overline{\hat{a}}(\xi+ \pi),\qquad\hat {b}_{2}(\xi)=\gamma(\xi)+e^{-i\xi}\gamma(-\xi), \qquad\hat{b}_{3}(\xi)=e^{-i\xi}\gamma(-\xi)-\gamma(\xi). $$

*Let* $\Psi=\{\psi^{1},\psi^{2},\psi^{3}\}$, *where* $\hat{\psi}^{j}(2\xi)=\hat{b}_{j}(\xi)\hat{\phi}(\xi)$, $j=1,2,3$. *Then* $X(\Psi)$ *is a tight frame for* $L_{2}(\mathbb{R})$. *Furthermore*, *the corresponding truncated operator* $\mathfrak{H}$ *provides approximation order* $2l+2$.

### Proof

The proof of tight frame is analogous to that of Construction 4.4 in [3]. We only give the proof of the approximation order. Firstly, we compute the order of zeros of $1- |{_{2}\hat{a}} |^{2}$ at the origin. Because $R_{r,m,l} (\sin^{2} (\frac{\xi}{2} ) )={_{2}\hat{a}_{r,m,l}}(\xi)$, we have

$$ 1- |{_{2}\hat{a}} |^{2}=1-R^{2}_{r,m,l} \biggl(\sin^{2} \biggl(\frac {\xi}{2} \biggr) \biggr). $$

Taking the first derivative of $1- |_{2}\hat{a} |^{2}=1-R^{2}_{r,m,l} (\sin^{2} (\frac{\xi}{2} ) )$ with respect to *ξ*, applying (3) and (2) of Lemma 2.1, we can obtain

$$\begin{gathered} 1-R^{2}_{r,m,l} \biggl(\sin^{2} \biggl( \frac{\xi}{2} \biggr) \biggr)' \\ \quad= -2R_{r,m,l} \biggl(\sin^{2} \biggl(\frac{\xi}{2} \biggr) \biggr)R'_{r,m,l} \biggl(\sin^{2} \biggl( \frac{\xi}{2} \biggr) \biggr) \biggl(\sin^{2} \biggl( \frac{\xi}{2} \biggr) \biggr)' \\ \quad=2 \biggl(\frac{r}{2}-m \biggr) \biggl(\cos \biggl(\frac{\xi }{2} \biggr) \biggr)^{r-2m-1}R_{m,l} \biggl(\sin^{2} \biggl( \frac{\xi }{2} \biggr) \biggr)R_{r,m,l} \biggl(\sin^{2} \biggl(\frac{\xi}{2} \biggr) \biggr)\sin \biggl(\frac{\xi}{2} \biggr) \\ \qquad {}+2(m+l){m+l-1\choose l}R_{r,m,l} \biggl(\sin^{2} \biggl(\frac{\xi }{2} \biggr) \biggr){\sin^{2l+1} \biggl( \frac{\xi}{2} \biggr)} {\cos ^{r-1} \biggl(\frac{\xi}{2} \biggr)}. \end{gathered}$$

Since $R_{m,l} (\sin^{2} (\frac{\xi}{2} ) )$, $R_{r,m,l} (\sin^{2} (\frac{\xi}{2} ) )$, $\cos (\frac{\xi}{2} )$ is equal to 1 when $\xi=0$, and $\sin^{2l+1} (\frac{\xi }{2} )$ has zero of order $2l+1$ at $\xi=0$, we conclude that

$$ 1- |{_{2}\hat{a}} |^{2}=1-R^{2}_{r,m,l} \biggl(\sin^{2} \biggl(\frac {\xi}{2} \biggr) \biggr)=O| \xi|^{2l+2}. $$

Since _2_ *ϕ* satisfies the Strang-Fix condition of order *r*, the corresponding truncated operator $\mathfrak{H_{n}}$ provides the approximation order $\min\{r,{2l+2}\} =2l+2 $, for $0\leq l\leq{m-1}$, $r\geq2m$. □

## Example

In this section, we give an example to illustrate our main result.

### Example

We choose *â* to be the mask of the smoothed pseudo splines of type II with order $(6,2,1)$, i.e.,

$$ \hat{a}(\xi)=\cos^{6} \biggl(\frac{\xi}{2} \biggr) \biggl(1+2\sin ^{2} \biggl(\frac{\xi}{2} \biggr) \biggr). $$

**Figure 1:**
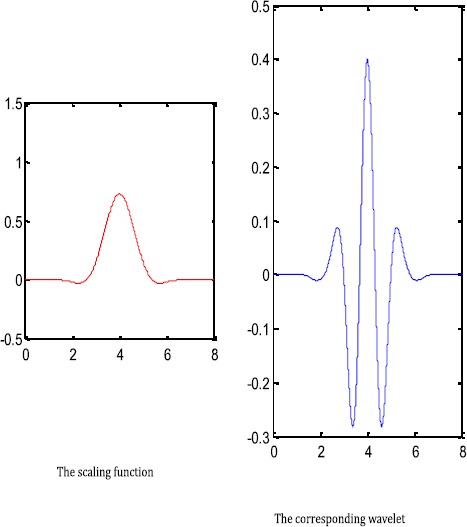
**Graphs of the scaling function and the corresponding wavelet.**

We define

$$ \hat{b}(\xi)=e^{-i\xi}\overline{\hat{a}(\xi+\pi)}=e^{-i\xi}\sin ^{6} \biggl(\frac{\xi}{2} \biggr) \biggl(1+2\cos^{2} \biggl(\frac{\xi }{2} \biggr) \biggr) $$

is a mask of the corresponding wavelet *ψ*. Then, by Theorem 4.1, $X(\psi)$ forms a Riesz basis for $L_{2}(\mathbb{R})$. The wavelet *ψ* has six vanishing moments. See Figure 1 for the graphs of the functions $\phi_{6,2,1}$ and *ψ*.

According to Theorem 4.2, if the refinement masks satisfy the factorized condition of Theorem 4.2, the corresponding masks of biorthogonal wavelets with infinite masks from smoothed pseudo splines will be obtained. By verifying the condition of the biorthogonal wavelets in Theorem 4.2, we can get that the corresponding biorthogonal wavelets *ψ* have six vanishing moments from a given smoothed pseudo spline $\phi_{6,2,1}$.

## Conclusion

In this paper, we constructed biorthogonal wavelets and tight framelets from a given smoothed pseudo spline. By analyzing the relevant knowledge of pseudo splines and wavelets, we found that the shifts of the smoothed pseudo splines are linearly independent. Based on the linear independence of the shifts of pseudo splines, we derived the construction of biorthogonal wavelets. By using the unitary extension principle, we constructed tight framelets with desired approximation order from a given smoothed pseudo spline.

It is interesting to note that in both theory and application, the family of wavelets and framelets studied exhibits good mathematical properties. In the future, we will consider how to achieve the construction of the compactly supported biorthogonal symmetric or anti-symmetric multi-wavelets and framelets with a dilation factor other than 2.
